# Untangling the complexity of multimorbidity with machine learning

**DOI:** 10.1016/j.mad.2020.111325

**Published:** 2020-09

**Authors:** Abdelaali Hassaine, Gholamreza Salimi-Khorshidi, Dexter Canoy, Kazem Rahimi

**Affiliations:** a Deep Medicine, Oxford Martin School, University of Oxford, Oxford, United Kingdom; b NIHR Oxford Biomedical Research Centre, Oxford University Hospitals NHS Foundation Trust, Oxford, United Kingdom; c Nuffield Department of Women’s and Reproductive Health, University of Oxford, Oxford, United Kingdom

**Keywords:** Machine learning, Deep learning, Multimorbidity, Electronic health records, Phenotyping

## Abstract

The prevalence of multimorbidity has been increasing in recent years, posing a major burden for health care delivery and service. Understanding its determinants and impact is proving to be a challenge yet it offers new opportunities for research to go beyond the study of diseases in isolation. In this paper, we review how the field of machine learning provides many tools for addressing research challenges in multimorbidity. We highlight recent advances in promising methods such as matrix factorisation, deep learning, and topological data analysis and how these can take multimorbidity research beyond cross-sectional, expert-driven or confirmatory approaches to gain a better understanding of evolving patterns of multimorbidity. We discuss the challenges and opportunities of machine learning to identify likely causal links between previously poorly understood disease associations while giving an estimate of the uncertainty on such associations. We finally summarise some of the challenges for wider clinical adoption of machine learning research tools and propose some solutions.

## Highlights

• Machine learning is making significant contributions towards our understanding of the complex relationships between diseases.
• Advanced models take a range of modalities from big datasets with little pre-processing or information loss at study design.
• Developments in matrix factorisation and deep learning allow a better understanding of evolving patterns of multimorbidity.

## 1 Introduction

**Fig. 1:**
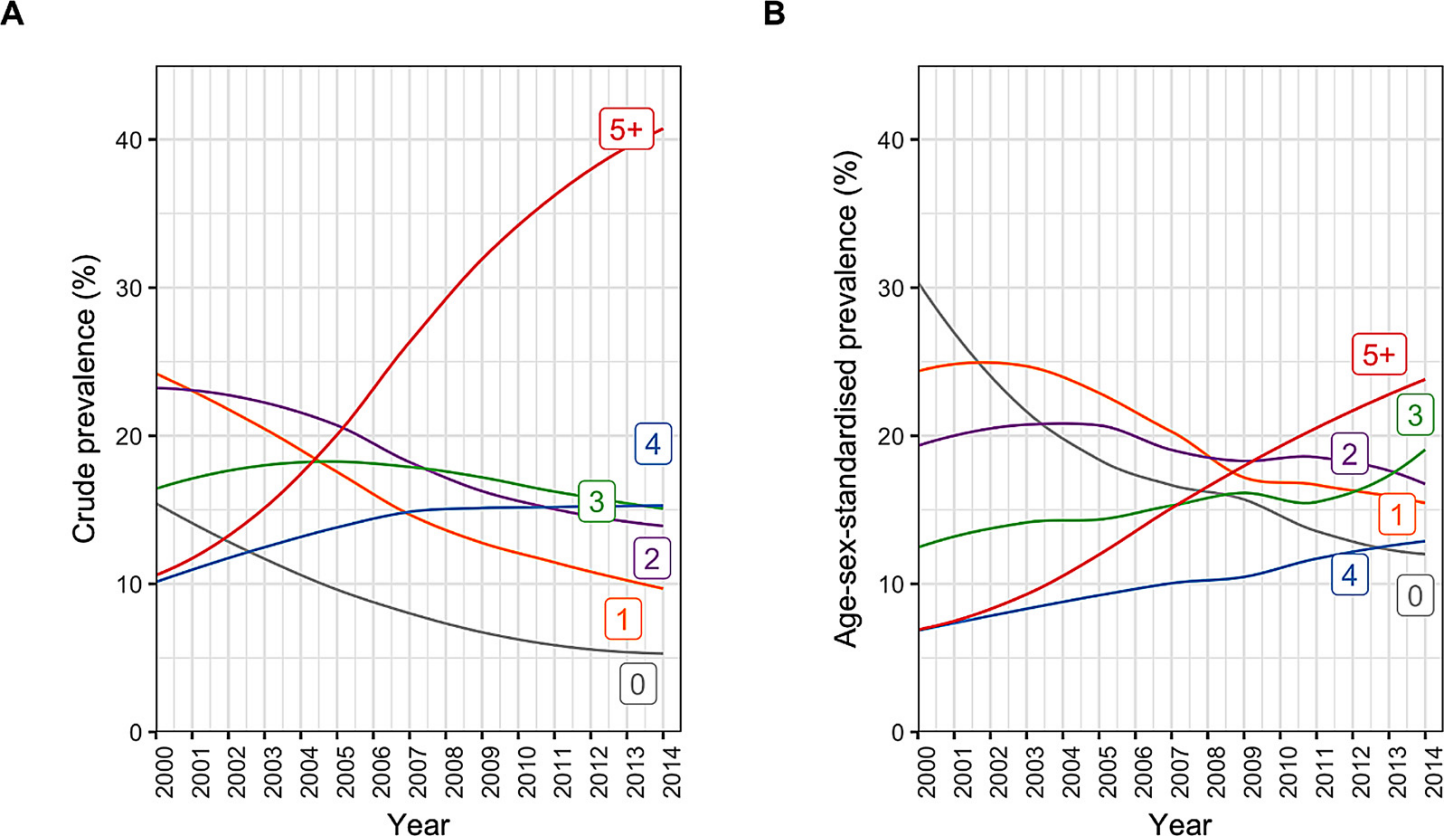
Annual crude and age/sex-standardised prevalence of number of comorbidities in incident cardiovascular disease patients (credits to Tran et al. (2018)); Number labels for each line refer to the number of comorbidities. (A) Crude prevalence. (B) Age/sex-standardised prevalence.

Advances in medicine have led to an increase in life expectancy and reduction of major disabilities. These achievements have also contributed to the rise in chronic conditions (that are more prevalent in older ages) and their co-occurrence, a phenomenon known as multimorbidity, that is, the simultaneous presence of two or more chronic conditions in the same individual) (The Academy of Medical Sciences, 2018). Indeed, research has shown that the proportional increase in multimorbidity over the past few years is only partially explained by population ageing, stressing its relevance to young and middle-aged adults (Fig. 1).

Medical research commonly focuses on the study of diseases – and their prevention and management – in isolation. Many such conventional approaches are likely to remain relevant to narrower questions relating to multimorbidity and testing of specific hypotheses, but are unlikely to be sufficient to answering questions relating to clustering of multiple diseases and their interactions as an important step towards identification of strategies for their prevention and management (The Academy of Medical Sciences, 2018).

Multimorbidity is characterised by a high degree of complexity arising from the presence of multiple diseases, their biological and non-biological determinants and consequences, and multiple interactions over time. Although such complexities are not unique to multimorbidity, they have not been sufficiently leveraged or embraced in prior research in the field. For instance, most previous studies of multimorbidity have been cross-sectional, which renders them unsuitable for the investigation and characterisation of how a disease progresses over time, taking into account how the trajectory interacts with its broader context (e.g., presence of other diseases, use of medications, and, more broadly, a patient’s entire medical history). Studies have often been based on small samples sizes or have focused on a small subset of conditions, hampering their ability to mine the disease clusters and phenotypes that are less frequent and, hence, poorly understood (The Academy of Medical Sciences, 2018). Thus, the complex temporal dynamics of multiple interactions inherent to multimorbidity has highlighted the importance of employing alternative methods that are better suited for tackling this complexity.

Outside multimorbidity research, one can draw parallels to the growing number of studies that aim to discover and characterise the so called “computable phenotypes” (Bennett et al., 2017), using various modalities of medical data that go beyond diagnoses, and considering additional information such as medications, interventions, physical measurements, and laboratory results. Most such studies aim to help the emerging field of precision medicine with the optimal care pathway for patients, based on their stratification into population subgroups that they have derived. Although faced with the similar challenge of complexity, the key difference to multimorbidity research is that such deep phenotyping studies have been aiming to define more homogeneous groups among patients with the same single diagnosis. Viewed from this perspective, the study of multimorbidity could be diagnosis-wide phenotyping, when multiple diseases within the same individual are considered simultaneously, or as comprehensive multi-modal phenotyping, when in addition to multiple diseases, information about the broader context in which diseases occur is also taken into account. In other words, complex multimorbidity modelling would ideally consider the entire medical history of an individual into account in an effort to reveal hidden patterns within the population without necessarily starting with a single condition.

Of course, the comprehensive discovery of such phenotypic classes (including, but not limited to multimorbidity classes) and their translation into clinical care, will depend on a number of constraints and choices – from study design and data availability, to the computational paradigms (or models) employed by researchers. In recent years, rapid developments in machine learning (ML), including deep learning (DL), have led to outstanding results (and at times superhuman performance) in previously difficult tasks, such as autonomous driving (El Sallab et al., 2020), machine translation (Devlin et al., 2018), computer vision (He et al., 2016), strategic decision making (Silver et al., 2017), and in domains with vast search spaces (Chen et al., 2016). Despite their relatively recent adoption in healthcare, ML methods have started to show promising results in drug discovery and development (Ekins et al., 2019), large-scale gene expression profiling (Chen et al., 2016) histopathological diagnosis (Litjens et al., 2016), brain MRI segmentation (Akkus et al., 2017), and disease prediction using electronic health records (EHR) (Ayala Solares et al., 2020; Hassaine et al., 2019; Li et al., 2020) (readers are referred to (Rajkomar et al., 2019) for a more comprehensive review of ML in medicine).

Given the importance of methodology in multimorbidity research, and the latest developments in the field of machine learning, this paper aims to review the state of relevant methodology and introduce some of the latest ML developments that have the potential to further advance this field. We will start in Section 2 by describing some of the key methodologies that have been employed for the study of multimorbidity (e.g., those that are based on network analysis and matrix/tensor factorisation). In section 4 we describe some of the methodological challenges and suggest potential solutions for them. In section 4 we will introduce some of the recent advances in matrix and tensor factorisation, deep learning, and topological data analysis. Despite their high potential for impact meaningful contribution to in the field, these latter methods have not yet been employed and evaluated for multiformbidity research. We will then conclude with summary of the approaches presented and suggestions for future research.

## 2 Current state of methodology

The field of multimorbidity research has already seen the use of a wide range of techniques for mining multimorbidity patterns - from network modelling, to probabilistic models and matrix (and tensor) factorisation techniques. This section provides an overview of how these methods have been applied in multimorbidity research.

### 2.1 Pairwise methods

Some of the earlier research using this method take an approach by initially assessing diseases as pairs and then combining the results across a wider range of diseases. In the pairwise class of techniques, disease pairs that show co-occurrence frequencies that are higher than their predicted individual frequencies in the population, are considered to be “connected”. In one of the early works in this category, Hidalgo et al. (2009) built a disease network in which the nodes and edges represented diseases and their connectivity, respectively. To overcome the challenge of missing temporal information in the resulting network, the authors carried out correlation analyses to decide whether a node property spreads along the links of the network and modelled how diseases propagate over time through the network. In another landmark study, Jensen at al. (Jensen et al., 2014) proposed a temporal disease network in order to provide pairwise methods with the ability to explicitly deal with time. In this approach, each edge represented a pairwise connectivity plus the time difference between the incidence of diseases that the edge connects. In a similar approach, Giannoula et al. (2018) used the pairwise connectivity plus the disease-timing data to cluster the diseases using dynamic time warping. The use of pairwise methods for mining multimorbidity patterns and phenotyping was not limited to disease data alone. (Goh et al. (2007) built a bipartite graph of genes and diseases, as a framework for the study of phenotype- and disease-gene associations.

While pairwise methods are valuable in generating comorbidity hypotheses for disease pairs, their inability to address conditional probabilities of multiple diseases directly (Pearl, 2009) can make the resulting multi-disease networks potentially misleading.

### 2.2 Probabilistic methods

Another class of models that have been employed for mining multimorbidity patterns can be referred to as “probabilistic methods”. Instead of simply looking at pairs of diseases, these methods provide a wholistic view of the relationships among diseases. For instance, (Strauss et al. (2014)) applied latent class growth modelling to a small UK EHR dataset, to identify clusters of multimorbidity trajectories. The authors clustered patients based on how many chronic conditions they developed over time, into 4 different groups ranging from no recorded chronic problems to increasing number of chronic morbidities. Although this work provided important insights about the accumulating number of diseases over time, it was not designed to assess the temporal relationships of diseases with each other or other patient covariates, which is a key aspect in the study of multimorbidity. Another approach to modelling multimorbidity trajectories is the use of computationally intensive Hidden Markov Models. Such models can learn progression of an individual’s health trajectory while incorporating time as a continuous variable. In an early example, (Wang et al. (2020a)) applied this method to patients with chronic obstructive pulmonary disease and showed how different patient groups developed additional comorbidities over time. The discovery of such distinct trajectories, as the authors argued, could assist decision makers to better understand the heterogeneity in disease progression and help researchers to potentially identify more targeted interventions. Although focused on the progression of a single disease, the modelling approach could potentially be applied for analysis of multiple disease trajectories over time.

### 2.3 Factorisation methods

Factorisation methods have seen a growing popularity in many fields including the study of multimorbidity. They have been extensively used to extract latent factors in many domains including image segmentation (Zhang et al., 2019) recommender systems (Abdi et al., 2018) and finance (Sun et al., 2016). Factorisation assumes that each patient’s medical record is the result of combining multiple “underlying factors” that are common across the population; the variability from one patient to another is due to the extent to which such factors are expressed in each patient, at each time/age. A factor can be thought of as a unique combination of concepts (such as diagnoses and medications) that can be found in EHR; for example, while one factor can denote ophthalmological disorders, another might be hypertensive diseases, and in patients with diabetes, both factors are likely to show a high expression. Factorisation allows the reduction of the multiple individual diseases or other features into a smaller set of factors that can explain the correlation between them.

**Fig. 2:**
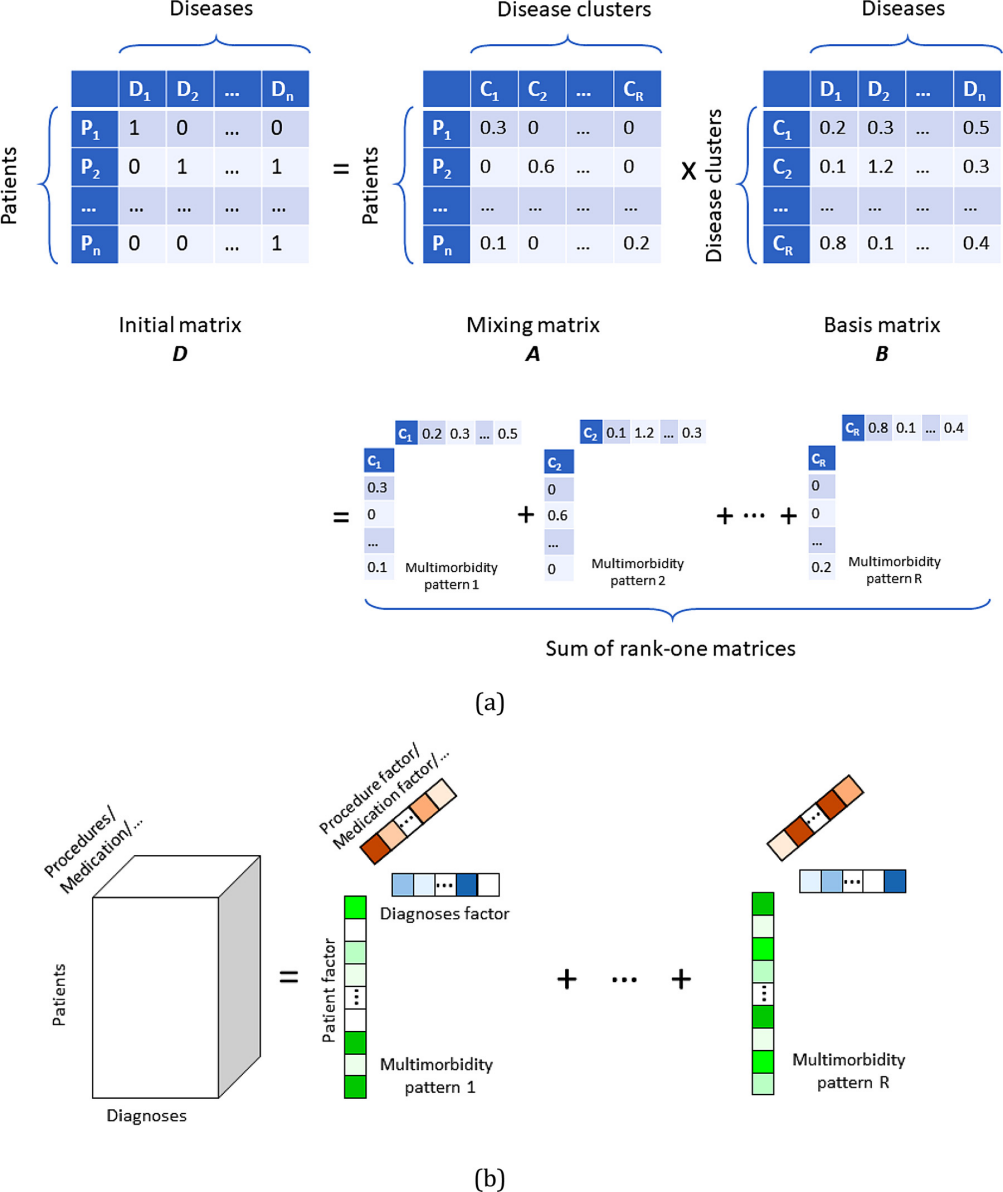
Two common types of factorisation methods employed in the multimorbidity literature; (a) Matrix factorisation, and (b) Tensor factorisation. Note that, one can change the concept that each dimension represents; in these illustrations, we show a very common way of choosing the dimensions.

In one of the simplest forms of these approaches, one starts by representing the data using a matrix ***D***, where patients and diseases are the two dimensions: ***D****(i,j) = 1* if patient *i* had disease *j* at some point in their life, and ***D****(i,j) = 0* otherwise. The factorisation is the process of decomposing ***D*** into two matrices ***A*** and ***B*** such that D≈A×B. The rank *R*, which is equal to the number of columns in **A** and the number of rows in **B,** is generally set through search and optimisation or based on empirical evidence. ***B*** is usually called the basis matrix, where each row *r* represents the belonging of every disease to the *r*’th component (i.e., the *r*’th disease cluster, or disease-based phenotype). ***A***, on the other hand, is called the mixing matrix, it shows how a linear combination of *R* clusters that can explain the diagnoses for each and every patient (see Fig. 2.a for an illustration).

This family of methods have seen a growing popularity in the field, as unlike previously described methods, they do not require much expert knowledge and hence have the potential to lead to novel discoveries. In addition, they are capable of incorporating a large set of information and different modalities at the same time. In one study, for instance, Holden et al. (2011) and (Kirchberger et al. (2012) applied matrix factorisation to extract multimorbidity patterns from self-reported diagnoses. Schäfer et al. (2010) applied factor analysis to extract multimorbidity patterns of elderly patients. Similar studies have also been proposed on different datasets, such as the work of Roso-Llorach et al. (2018) who concluded that clusters of diseases obtained from an older patient cohort with multimorbidity using hierarchical cluster analysis and exploratory factor analysis were not always similar. The authors suggested factor analysis to be more useful for analysing multimorbidity patterns whereas hierarchical cluster analysis (a more conventional statistical approach that assigns diseases to different clusters) could serve in generating new hypotheses for inter-cluster and intra-cluster associations.

The aforementioned factorisation studies only considered diseases when forming ***D***, and hence cannot untangle the relationships that other clinical concepts (such as medications, procedures, or lab tests) could have on the natural history of the disease. In an attempt to alleviate such issues, Ho et al. proposed to add procedures as a third dimension (Ho et al., 2020), and Wang et al. added medications (Wang et al., 2020b); this process will change the problem from matrix factorisation (i.e., with a 2D input) to tensor factorisation (i.e., with a 3D input) – see Fig. 2.b for an illustration.

The methods described above produced factors that show disease-disease associations, as well as associations among diseases and other clinical concepts (such as medications and procedures), but as they did not account for temporal evolution of these factors over time, their clinical usability remains somewhat limited.

### 2.4 Temporal phenotyping

To account for the temporal aspects of multimorbidity, Zhou et al. (2020) represented each patient’s EHR using a matrix, where the two dimensions were diagnoses and time. One of the strengths of their approach is its ability to handle missing data, which is common in EHR. The authors showed that the obtained phenotypes are useful in predicting the onset of new diseases, such as congestive heart failure or end stage renal disease. However, the clinical relevance of the derived phenotypes remains uncertain. In a different approach, Perros et al. (2019) considered the chronological order of encounters (as opposed to the time/age at which they actually happened). Their method has recently been extended by Afshar et al. (2019) to jointly account for dynamic and static information (such as demographics information). The authors showed that this method produces clinically meaningful phenotypes that yielded accurate heart failure prediction, but given that the intervals between two consecutive encounters can contain important medical information and vary greatly from patient to patient (and even for the same patient), explicitly accounting for the time in these methods – as opposed to order – can be a natural improvement.

**Fig. 3:**
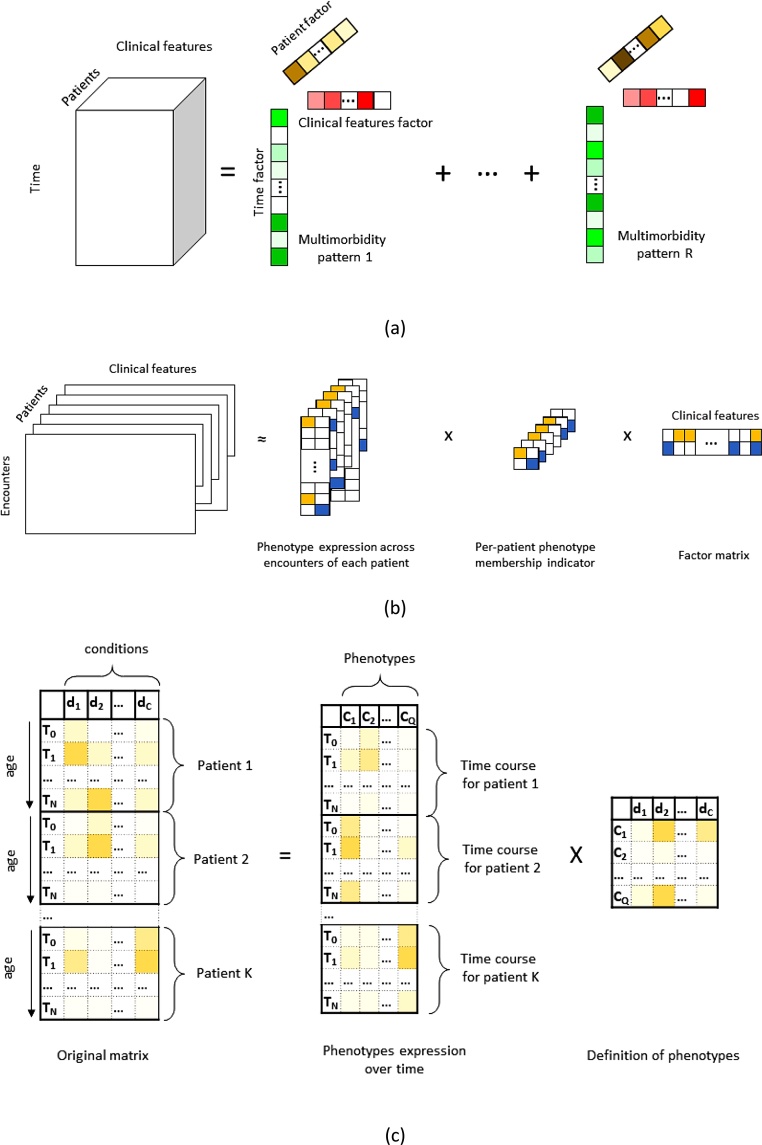
Examples of temporal phenotyping. (a) using a tensor where time is mapped to a dimension, (b) using a tensor where the encounters are mapped to a dimension (c) using concatenated matrix representations.

In another study, (Zhao et al. (2019)) incorporated the time to the onset of cardiovascular disease as a dimension in the tensor. The phenotypes obtained from this approach, while temporally profiled, are specific to cardiovascular disease patients (see Fig. 3.b for an illustration of this method).

**Fig. 4:**
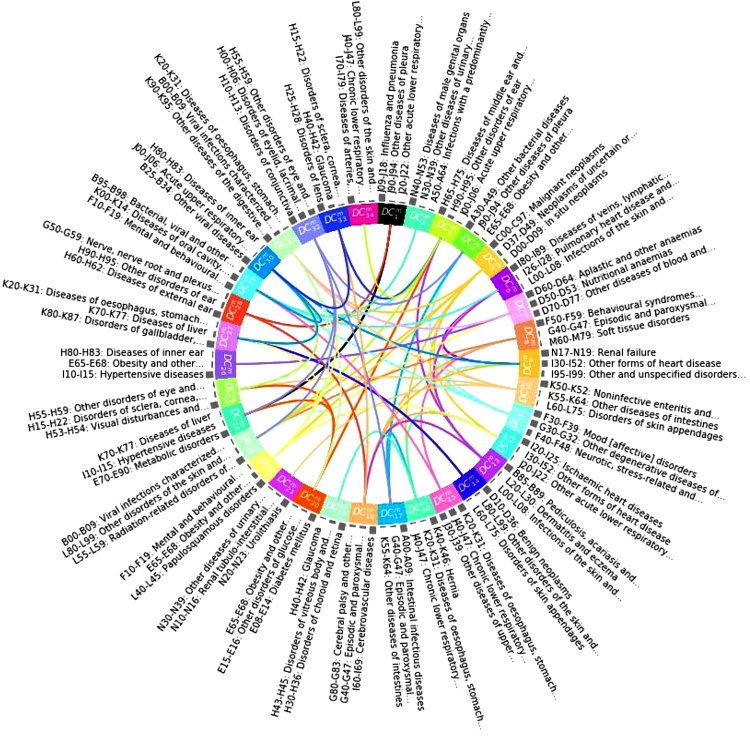
Network showing how male disease clusters (DC) may lead to one another over time. Edges are coloured with the colour of the nodes they originate from. Credits to (Hassaine et al., 2019).

In another study that looked at all recorded primary and secondary care diagnoses, matrix factorisation was used to temporally profile the multimorbidity clusters (Hassaine et al., 2019). In this approach, each patient’s EHR results in a matrix; instead of representing patients in a third dimension, it concatenates the patient matrices along the time dimension and, hence, stays in the matrix domain (Fig. 3.c). The authors showed how the temporal characteristics of the disease clusters that result from this model could help mine multimorbidity networks and generate new hypotheses on how multimorbidity patterns may lead to one another over time. (Fig. 4)

As a complementary material to this section, Table 1 provides a non-exhaustive list of recent multimorbidity studies that were not cited in this section but have introduced new methods that fall within one of the four categories we mentioned earlier.

**Table 1:** A summary of the papers that introduced a new method for the study of multimorbidity patterns.

Study	Method	Context	Data
Hernández et al. (2019) (Hernández et al., 2019)	Pairwise correlations	6101 Irish adults aged 50+ years	Self-reported conditions
Aguado et al. (2020) (Aguado et al., 2020)	Pairwise correlations	500 K adults in Spain with Type 2 diabetes mellitus.	EHR
Jin et al. (2018) (Jin et al., 2018)	Pairwise correlations	21,435 adults from Jilin province, China	Self-reported conditions
Khorrami et al. (2020) (Khorrami et al., 2020)	Latent class analysis	10,069 adult Iranian people	Self-reported conditions
Wang et al. (2019) (Wang et al., 2019)	Principal Component Analysis	2713 adults in São Paulo, Brazil	Self-reported conditions
Schiltzet al. (2017) (Schiltz et al., 2017)	Classification/regression trees and random forest	5771 people from US aged 65+ years	Self-reported conditions linked to Medicare claims
Haug et al. (2020) (Haug et al., 2020)	Hierarchical clustering	5M patients in Austria	EHR
Bueno et al. (2018) (Bueno et al., 2018)	Hidden Markov Models	Dutch patients with comorbidities related to atherosclerosis	EHR
Violán et al. (2018) (Violán et al., 2018)	K-means non-hierarchical cluster analysis	400 patients aged 45−64 years from Spain	EHR
Marengoni et al. (2019) (Marengoni et al., 2019)	Fuzzy c-means cluster algorithm	2931 individuals in Sweden aged 60+ years	EHR
Medlock-Brown et al. (2019) (Madlock‐Brown and Reynolds, 2019)	Pairwise correlations	574,172 patients with obesity in the US	EHR

## 3 Methodological challenges

There are a number of areas where further application of methodological developments can be beneficial; this section aims to discuss a few of these challenging areas and share ideas on how to potentially tackle them.

### 3.1 Big, comprehensive, and longitudinal data

**Fig. 5:**
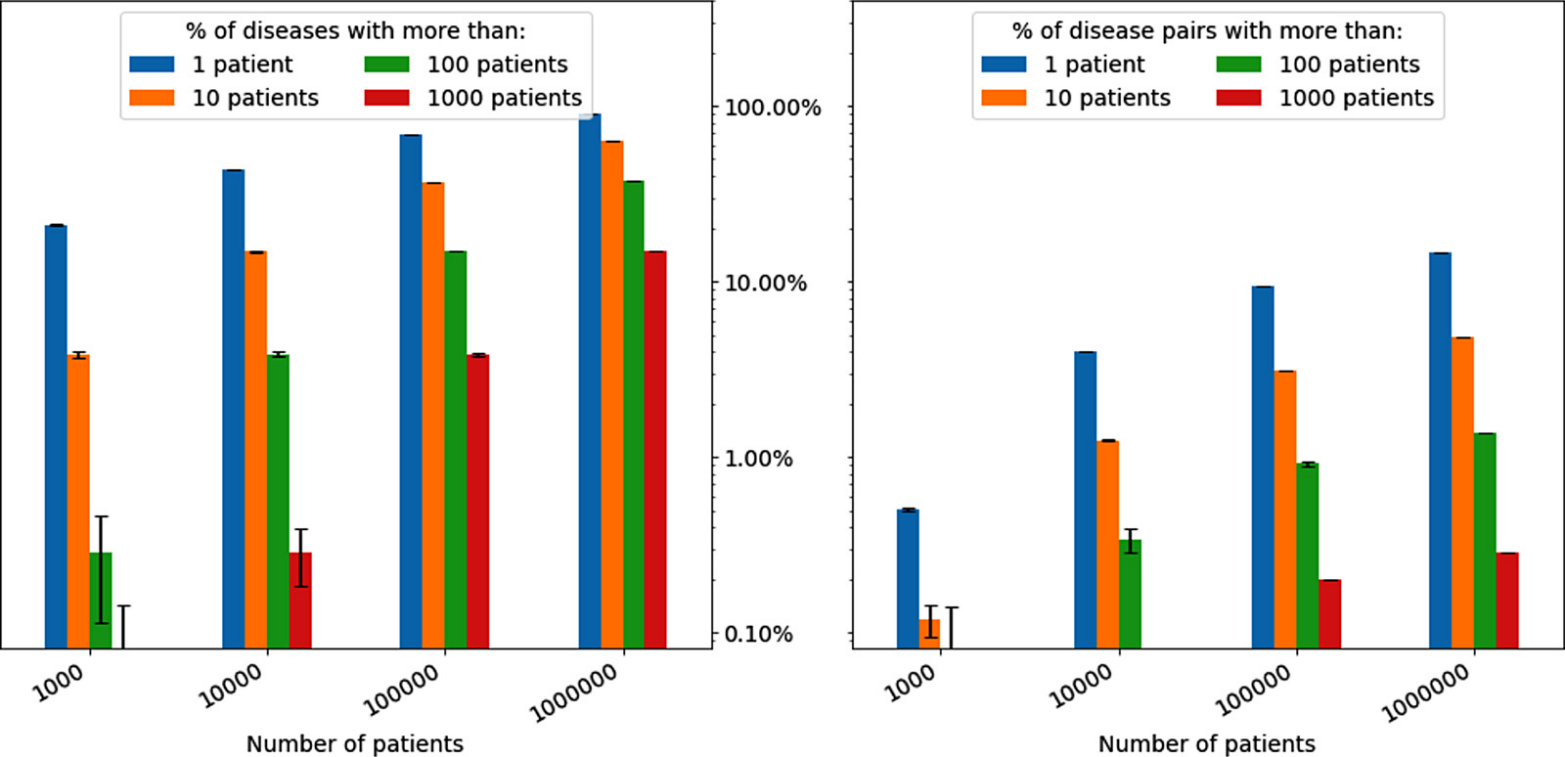
Occurrence and co-occurrence of diseases as a function of the size of the dataset, error bars correspond to 95 % CI estimated using 10 bootstrapped samples. Experiments conducted on CPRD (Herrett et al., 2015).

Characterising each disease’s dynamic relationship with its context could be very informative, hence, the consideration of a more comprehensive range of concepts or modalities in studies of multimorbidity should be relevant. However, the number of all possible relationships in such data is of a super-exponential size with respect to the number of concepts in the data, so the feasibility of handling such large data needs to be considered. For instance, in ICD-10 coding scheme, there are nearly 9800 diseases at the 4-character level; nearly 96 % of these diseases have a prevalence of less than 1%. On the other hand, multimorbidity analysis is interested in *co-occurrences*; for less frequent diseases, their co-occurrences are likely to be even rarer. Therefore, the larger the sample size (or, N), the more likely for the data to include more cases of occurrences and co-occurrence of many diseases (see Fig. 5).

**Fig. 6:**
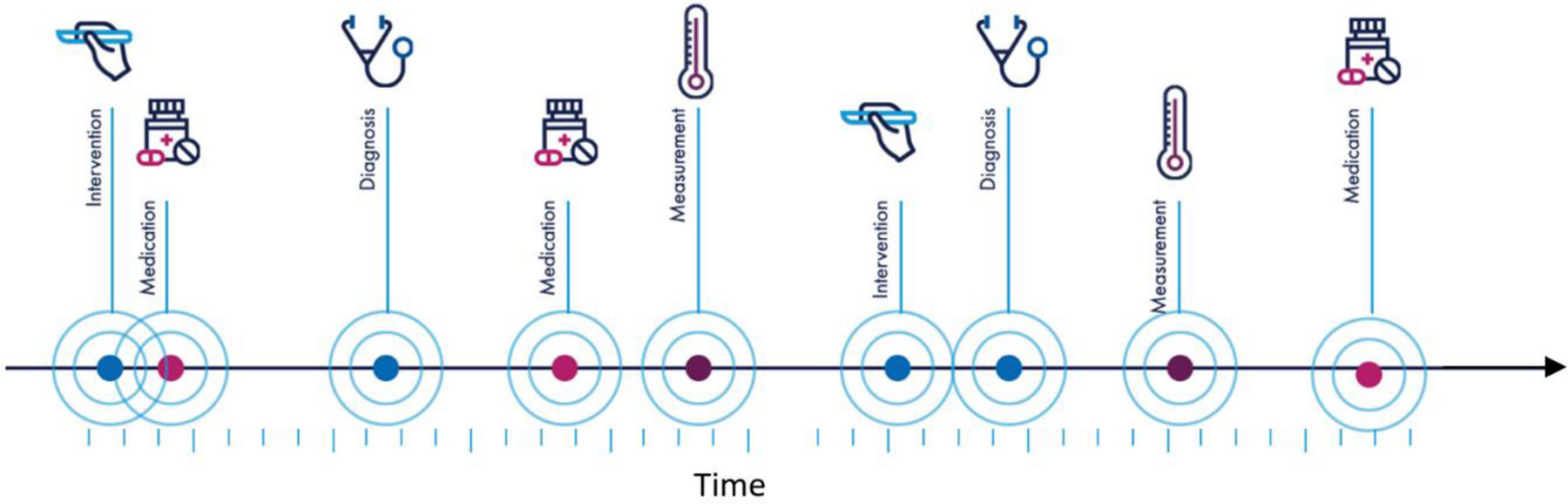
Illustration of a patient’s multimodal health record, where events/encounters tend to happen at irregular intervals.

One of the ideal sources of such large-scale data are EHR, which contain mixed-type multimodal sequences of concepts, that occur in irregular intervals (see Fig. 6). When dealing with EHR data, one should also consider practical challenges. One such challenge is the skewed distribution of disease frequencies (incidence or prevalence). This means that any modelling technique aiming to explain the occurrence and cooccurrence of diseases in patients’ EHR, is likely to be biased towards more frequent diseases. Methods such as DF-IPF (disease frequency, inverse patient frequency) (Hassaine et al., 2019) have been suggested which aim to alleviate this risk. Inspired by a commonly used technique in information retrieval (when dealing with rare words), DF-IDF multiplies the frequency of each disease by the “*inverse patient frequency”* factor, which assigns higher weights to rarer diseases and can be particularly useful for studies that aim to discover new mechanisms of disease propagation (as opposed to description of absolute risks or frequencies in populations).

Another practical aspect of using routine large-N data such as EHR is the time window it covers for each individual. Many disease co-occurrences and interactions take years to develop whereas others have a more immediate effect on each other. Therefore, in order to draw meaningful conclusions such as disease A leads to disease B, longitudinal properties of the data are extremely important and one needs to choose a dataset that has multiple years of follow up for a large number of patients. Finally, it is important to note that most EHR datasets were established for clinical, administrative or audit purposes and, as a consequence, such datasets may have missing information, be subject to ascertainment bias, or contain inaccurate or erroneous information, hence the need to be adapted and ‘transformed’ for research purposes (Nissen et al., 2019).

### 3.2 Incorporation of prior knowledge

Medicine often accumulates (and updates) its knowledge-base over several centuries, thus, developing areas of expertise and specialist knowedge. On the other hand, one of the greatest advantages of ML has been its minimal reliance on domain expertise (e.g., automatic feature engineering). In recent years, however, multiple studies have shown how an optimal combination of domain knowledge and data-driven ML can lead to superior results – better than each of them individually (K M et al., 2020). For instance, one of the challenges in multimorbidity research is the preparation of the input data (e.g., matrix or tensor), which leads to questions such as, ‘*Will diseases only be assigned to patients through disease codes, or can certain medications and test results in the record also indicate a diagnosis*?”, “*Which disease coding scheme should one use (e.g., ICD-10, Read Code, SNOMED) and at what level of granularity?*”, “*How should one consider the incidence, recurrence, or recovery and cure from diseases?*”, or “*Should one denote and deal with chronic diseases differently, compared to acute conditions?*”.

These are just a few example questions that a researcher might face at the start of their modelling work, which is likely be helped with some level of the medical knowledge. This issue is highlighted, for example, when making a distinction between chronic and acute conditions; available annotations, such as the Chronic Condition Indicator (Research, U.S.A.f.H), could be used. Given that multimorbidity studies tend to focus on chronic conditions, filtering of the information accordingly might be appropriate.

Multimorbidity studies represent patients differently in many aspects, which greatly hampers their ability to compare their results. Defining diseases may be as granular as 3 or 4 characters of ICD-10 codes or as broad as ICD-10 blocks, CALIBER codes (Kuan et al., 2019), Clinical Classification codes (Elixhauser et al., 2014), Hierarchical Condition Category (Abdi et al., 2018) or SNOMED-CT codes (Donnelly and informatics, 2006). Patients may also be represented based on their other clinical features, and standardised approaches could be useful. Time has also dealt with in different ways as mentioned earlier (see section 2.4), efforts are still needed to reach a unified way for modelling the temporal aspect.

Open-sourcing code related to multimorbidity research contributes greatly toward making different methods comparable, it is encouraging to see that many scholars in the field are making their code available (Li et al., 2020; Beam et al., 2020; Choi et al., 2016a; Cai et al., 2020). The use of standard data models such as the Observational Medical Outcomes Partnership (OMOP) (Stang et al., 2010) is likely to result in a wider clinical adoption than internal data models which are often based on ad-hoc local decisions on how to store, load and represent the data. Studies that compare different methods on the same dataset are also important to tackle this issue, although first efforts have been made to assess the predictive power of different methods (Ayala Solares et al., 2020; Barbieri et al., 2020), further research is needed to compare multimorbidity patterns and trajectories derived from different models. Finally, further work is needed in order to establish the best practices for ML related research in multimorbidity.

### 3.3 Evaluation of results

Multimorbidity research has usually been carried out as an unsupervised learning task, in which conclusions are drawn from models which are not labelled with the outcome of interest. This will make the quantitative evaluation of the goodness of the results a big challenge; it will rather rely on manual profiling and qualitative assessments, and, hence, risks the influence of subjective options.

A potential solution for such quantitative assessments can emerge from translating medical texts (in areas such as comorbidities) to knowledge graphs and other formats that can be used to quantify the correspondence between the mined relationships and well-established medical knowledge. In an example work in this area, Beam et al. compiled a set of disease pairs which are either known to be associated or as having a causal relationship (Beam et al., 2020). In another study (Hassaine et al., 2019), authors used these disease pairs to evaluate the clinical meaningfulness of multimorbidity patterns that they derived from the data. Furthermore, they extracted another set of comorbidity pairs from a Danish EHR study as a clinically accepted reference (Jensen et al. (2014)) to quantitively compare their results.

## 4 Emerging ML opportunities

Despite the diversity of methods that the field has employed so far, there are many new developments in ML that have not yet been used for the study of multimorbidity. These methods have the potential to improve the state of methodology research in multimorbidity. In this section, we provide an overview of such opportunities and share ideas as to how the field can benefit from them.

### 4.1 Alternative factorisation techniques

Most factorisation methods used in the multimorbidity research are related to Non-negative Matrix and Tensor Factorisation approaches (i.e., NMF and NTF); both techniques take a non-negative input and factorise them into non-negative matrices, for which the multiplication should reconstruct the original input. The resulting patterns, due to non-negativity, are pushed to be sparse and, therefore, easier to interpret (Hassaine et al., 2019; Ho et al., 2020; Wang et al., 2020b; Afshar et al., 2019; Zhao et al., 2019). However, we know that some diseases are likely to have a suppression (negative) effect on each other; diseases A and B can be said to have suppressive effect on each other if the presence of disease A leads to lower risk (or prevention) of disease B, perhaps because of shared risk factors and clinical management (e.g. treatment of disease A has also some clinical benefit to disease B, or assessment of disease A also identified disease B) (Lagu et al., 2008; Magnan et al., 2015; Min et al., 2007). The non-negative factorisation techniques, while effective in mining coincidence patterns (e.g., where each disease belongs to disease clusters with a *non-negative* membership score), are not able to mine such suppressive patterns (e.g., where each disease belongs to disease clusters with either a *negative or non-negative* membership score). In addition to disease clusters, a similar effect can exist for their expressions, i.e., the expression of a particular disease cluster in a patient, can reduce the risk of the expression of another disease cluster. Independent Components Analysis (ICA) is an example factorisation technique that not only can mine disease clusters that are statistically independent (plus their corresponding expressions), but also has the potential to learn both co-occurrence and suppression relationships (Hyvärinen, 2013). It has been successfully applied in fields such as magnetic resonance imaging (fMRI) to help model spatial activation and deactivation maps (Beckmann and Smith, 2004), or as feature extraction method on gene expression data (Mollaee and Moattar, 2016).

Another important property that can be added to factorisation methods is their probabilistic implementation. For instance, when considering a disease cluster resulting from a non-probabilistic implementation of ICA or NMF, one cannot say if a particular disease’s score for a given cluster is significant (i.e., stronger than what can occur by chance alone); this hampers the field’s ability to define an effective null model or hypothesis, under which the numbers resulting from the factorisation methods can be evaluated. There are multiple probabilistic implementations of commonly used factorisation techniques (e.g., PCA, ICA, NMF), and the field can benefit from these implementations in order to estimate the statistical significance of the obtained associations. One such technique is the probabilistic ICA, s a common approach for exploratory analysis of MRI data where one can map the coefficients to Z-stats and probabilities (Beckmann and Smith, 2005).

In addition to hypothesis testing, and going beyond dealing with non-negative values, recent developments in deep learning (DL) have led to improvements in matrix and tensor factorisation methods. Chen et al. used an attention mechanism to combine several implementations of NMF for breast cancer prognostication using gene expression and clinical data (Chen et al., 2019). In another study, Schreiber et al. proposed a multi-scale deep tensor factorisation for learning latent representations of the human epigenome that shows a promising performance in both imputation and prediction tasks (Schreiber et al., 2019). Many of such deep matrix and tensor factorisation techniques have shown superior results when compared to their more traditional counterparts so their use in multimorbidity research has the potential to improve the results. In particular, when considering multimorbidity analysis as a multi-modal phenotyping, which is expected to result in temporal patterns (i.e., multi-modal temporal phenotyping) techniques such as temporal regularised matrix factorization (Yu et al., 2016) can provide a unique opportunity. In this technique, one can define a regulariser such as AR (autoregressive model) or GP (Gaussian process), which will force the time courses resulting from the factorisation to have certain properties that are aligned with our prior knowledge (e.g., that disease clusters emerge smoothly over time, rather than suddenly going on and off).

### 4.2 Representation learning

Unlike traditional ML, where one needs to represent the inputs by a number of expert-defined features (or markers), DL models have shown tremendous success in learning useful representations from raw or minimally processed data across layers of neural networks (hence, the names “representation learning” (Bengio et al., 2013) and “distributed representations” (Hinton, 1986)). They achieve these representations by using many linear and nonlinear transformations of their inputs, across multiple layers of neural networks. For example, one can use such techniques to learn new representations for individual medical concepts (such as diseases or medications (Choi et al., 2016b)) or a sequence of medical concepts (e.g., patient2vec (Zhang et al., 2018), cui2vec (Beam et al., 2020)). There have been multiple studies that showed the usefulness of such new representations for medical tasks, such as generating representations of biological sequences (Asgari and Mofrad, 2015), or extraction of insights about possible disease associations (Gligorijevic et al., 2016).

**Fig. 7:**
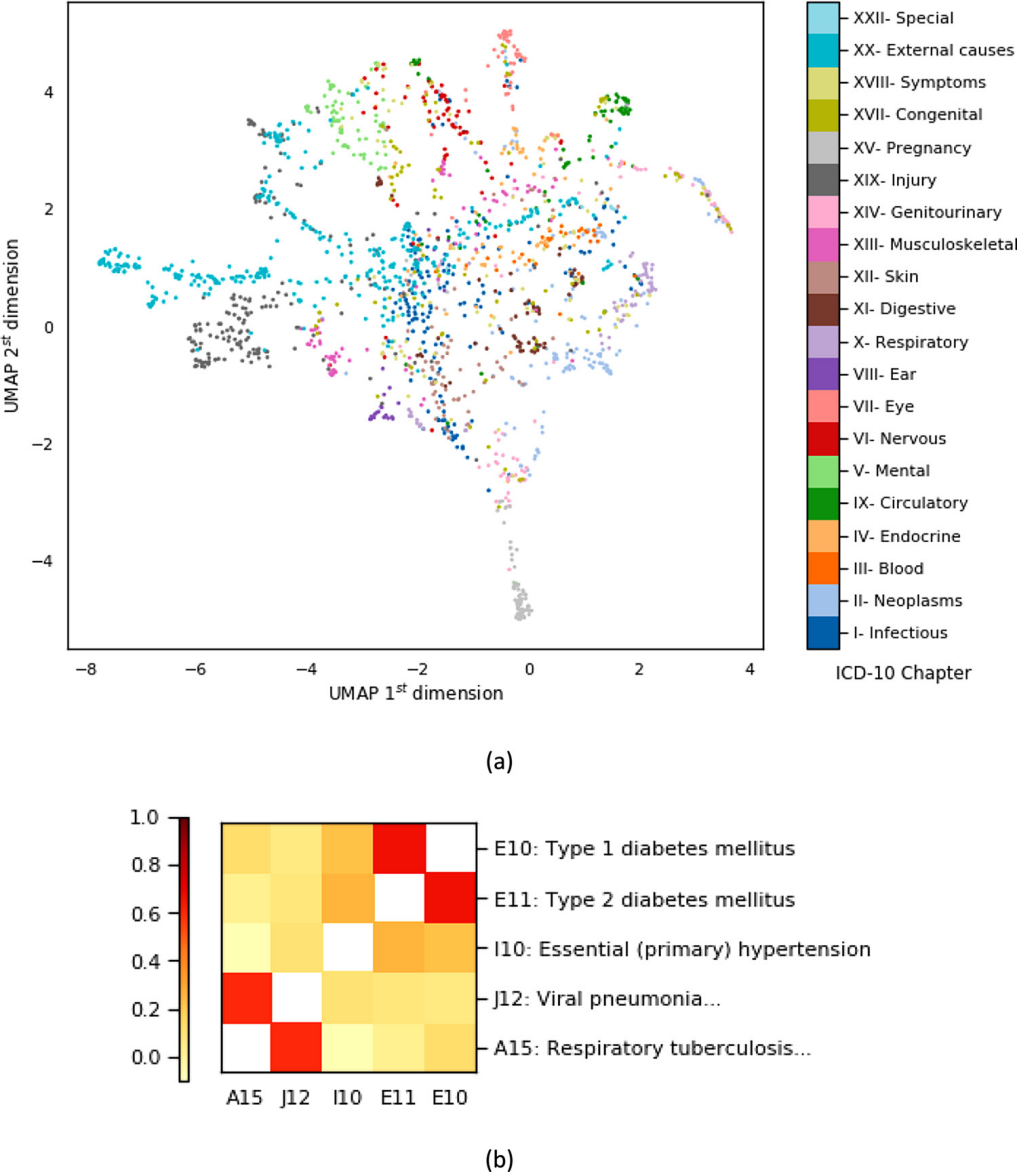
(a) UMAP projections of ICD-10 disease embeddings extracted using CBOW algorithm from CPRD. Note that diseases within the same ICD-10 chapter are very close in the embedding space. (b) Cosine similarity between vector embeddings of a selected group of diseases.

The learned representations resulting from such models can help map medical concepts such as diseases and medications to vector spaces, in which algebraic operations (such as computing similarity) can be carried out. For instance, word embeddings (i.e., learning vector representations of words) has enabled the field of Natural Language Processing (NLP) to learn the relationship among words given the context, and use this learning to generate sentences, and perform human-like (or at superhuman level) in various other NLP tasks; the famous anecdote “king - male + female = queen” (Mikolov et al., 2015) is one of the best examples to explain this concept. To illustrate the potential of such methods, we show in Fig. 7.a the Uniform Manifold Approximation and Projection (UMAP) (McInnes et al., 2018) of Continuous Bag of Words (CBOW) embeddings (Mikolov et al., 2013) extracted from the UK EHR (Herrett et al., 2015). Using such approaches, one can find disease clusters which may be known to medical experts or could be new. Furthermore, the distance calculated in these vector spaces, can be a useful approach to mine comorbid diseases from the data (Beam et al., 2020). For instance, Fig. 7.b, shows cosine similarities of some of the disease vectors that result from the same CBOW analysis. Note that the cosine similarity between similar/related diseases, such as type 1 and type 2 diabetes, is much higher when compared to that of *a priori* known to be unrelated diseases such as diabetes and pneumonia.

Another useful development in representation learning is transfer learning: exploiting commonalities between different learning tasks to transfer knowledge from one task to another (Tan et al., 2018). This can be especially helpful in populations and fields where large-N data are not easily accessible or when training requires big computational resources. In such cases, one can learn the likes of disease embeddings in an available large-N data and assess how diseases sequences lead to certain other disease/outcomes in the small-N data. Furthermore, transfer learning has the potential to unravel multimorbidity in rare diseases which are often under-investigated due to the lack of data availability and to generalise the learned multimorbidity patterns from a large population dataset to smaller ones.

The field of deep representation learning has seen many other developments in recent years. For the rest of this section, we will focus on the development of deep generative models (e.g., generative adversarial networks, or GANs), and attention-based sequence models. When appropriately trained on real-world data, GANs can generate realistic (but simulated) data, which will have similar properties to the data they were trained on. For instance, GANs have been successfully trained to generate realistic EHR data (Choi et al., 2020); such a development has the potential to create dynamic contexts that over time will lead to the appearance of certain disease clusters (i.e., multimorbidity trajectories). Furthermore, they can alleviate the previous obstacles in data sharing and patient privacy, which might have slowed down the research in this field and hampered its ability to use large-N data. However, models reported are still in their infancy and their suitability for modelling multimorbidity at scale remains to be further explored.

**Fig. 8:**
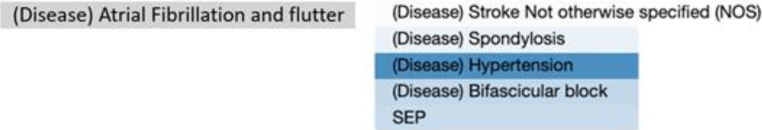
Illustration of the self-attention in BEHRT. The left column shows the outcome of interest, the right column shows the corresponding associations that “attracted” the attention of the model, the darker the colour, the more relevant was the disease in predicting the outcome of interest.

Given the sequential nature of disease progression, multimorbidity trajectories, and patients’ records, one can employ the latest developments in this field and the latest developments in deep sequence models, neural networks with attention mechanism can be another great area of exploration for the field. Attention mechanisms, for instance, allow the network to take into account past events as it sees relevant, without giving recent events a higher weight that is commonly implemented in traditional sequence models, such as Recurrent Neural Networks (RNN) and Long Short Term Memory (LSTM). This is extremely useful in medicine, where we know of many long-range dependencies: an operation in young ages might prevent one from being given a treatment in their older ages. For instance, an early example of such models for EHR data (Li et al., 2020), showed how the prediction of diseases in future can be accurately achieved through attention mechanism; mining diseases that might co-occur in a patient’s life-long medical history. Fig. 8 illustrates the attention mechanism in BEHRT by showing the diseases for which the model paid a particular attention (right column) when predicting the outcome of interest (left column).

### 4.3 Interpretable and causal ML with uncertainty estimation

While the use of ML has provided various fields, including medicine, with great accuracy in various prediction tasks, trust in ML and its use in action will require a higher degree of interpretability in some use cases. Topological data analysis (TDA) is an area of mathematics that has shown a great degree of synergy with ML. TDA provides mathematical, statistical and algorithmic methods to infer, analyse and exploit the topological and geometric structures underlying the data. The early use of TDA has led to a promising start; it has been used for unsupervised clustering of sub-phenotypes of conditions, such as diabetes and breast cancer (Li et al., 2015; Nicolau et al., 2011); it has also been used for tracking resilience to infections in mice by mapping their disease space (Torres et al., 2016). These studies show that TDA has the potential to unravel further relationships between diseases and offer an alternative representation of multimorbidity and contributes towards a better understanding of its patterns. TDA methods can be applied on the top of other ML techniques in order to produce visual outputs that can greatly contribute towards the interpretability of the results and hence build trust in the system.

As step further from interpretability is to investigate causal relationships (or mechanisms); this is an important goal for ML models’ success in medicine, where clinical evidence has been informed by well-conducted studies, such as randomised clinical trials. Causal inference from observational data is often a controversial issue (Collins et al., 2020) but many developments in the field have shown to be successful in tackling some of its limitations, such as the causal Bayesian networks which have been used to model disease progression mechanisms (Koch et al., 2017) or predict disease complications (Yousefi et al., 2020). Causal inference tools are also used to analyse the relationships between the learned deep learning phenotypes and patient outcomes and diseases of interest (Kale et al., 2015). Other interesting developments in the field include the introduction of causal effect variational autoencoders which make use of proxy variables as well as latent variable models for estimating individual and population causal effects (Louizos et al., 2020), the use of matrix factorisation to infer confounders from noisy covariates (Kallus et al., 2018), the use of embeddings to correct for confounding in networks (Veitch et al., 2019) and the analysis of networks of disease clusters to understand how multimorbidity patterns may lead to one another (Hassaine et al., 2019). All these developments can contribute towards distinguishing between possible causal and non-causal disease associations.

Lastly, most DL models are not good at estimating uncertainty and providing well-calibrated prediction; this property may lead to overconfident predictions in case of a dataset shift or a distributional change (Amodei et al., 2016). Therefore, finding a solution to assign a degree of uncertainty to their predictions will be an important step in making them usable in real-world clinical settings. In recent years, there has been multiple solutions that introduced the use of dropout Bayesian approximation (Gal and Ghahramani, 2020), Gaussian processes (Liu et al., 2020; Snelson and Ghahramani) and other improvements; some of these techniques have been used for uncertainty estimation in predicting trends in temporal disease networks using EHR data (Gligorijevic et al., 2018).

## 5 Conclusions

The field of medical research is showing a growing interest in understanding diseases in their context; while most such studies have defined the context as how diseases occur with respect to other diseases, one can take this further and employ the full richness of modern large-N data advanced ML techniques to define context as a multi-modal concept. Particularly, given the growing prevalence of multimorbidity in various populations, the result of such research can help the field improve our understanding of disease pathways and the range of approaches for treating them.

In this article, we reviewed a broad range of techniques that have been used, for both disease-only and multi-modal phenotyping; we highlighted the importance of accounting for the temporal information and showed how ML helps extract insights about the temporal evolution of multimorbidity patterns. We also discussed how considering all diseases at once instead of pairwise correlations yields more generalisable results and stressed that handling large quantities of data from different modalities, including drugs and procedure, is important for obtaining more informative phenotypes. We also shared ideas on how ML or DL models can provide us a range of solutions to go beyond cross-sectional, expert-driven and confirmatory approaches, and gain a better understanding of evolving patterns of multimorbidity that is hidden in EHR sequences.

Furthermore, we listed some of the latest trends in ML in both matrix/tensor and deep representation learning that have the potential to further unravel the complexities underlying multimorbidity; while factorisation methods tend to be easier to interpret, deep learning approaches have shown an unprecedented level of improvement in performance of medical predictive models. As explained in the paper, some of such deep neural models can be employed to mine the relationship between diseases and their broader context, e.g., using attention mechanism. Generative adversarial networks can be employed to generate realistic EHR datasets alleviating previous obstacles in data sharing and patient privacy; a combination of Bayesian ML/DL and causal ML/DL can help estimate uncertainties in models’ results and help with mining truly causal relationships, which in the long run can lead to a broader clinical adoption. Topological data analysis, on the other hand, has been shown an effective way to extract the topological and geometric structures underlying the data, which through its visual interrogation tools, can enable a broader group of health researchers to scrutinise the results and generate new hypotheses. Lastly, other techniques such as probabilistic factorisation can help distinguish between spurious and significant disease associations (i.e., co-occurrences that can happen by chance alone vs those that are truly meaningful); adding temporal regularisation to matrix factorisation techniques can provide a way to take into account temporal autocorrelations in diseases’ relationships with each other and their expression over time (and in various ages). All these methodological advances have a high potential to be used in mining computable phenotypes and multimorbidity patterns, but have yet to be fully explored and applied in multimorbidity research.

In addition to improving the state of data analytics, there are additional challenges that the field is still facing: data preparation and processing, incorporation of prior expert-knowledge, and mapping the final results to clinical use and medical guidelines. The last point can potentially be improved by the introduction of techniques that can evaluate such models’ results by comparing against existing clinical knowledge.

The funding organizations had no role in design or conduct of the study; collection, management, analysis, and interpretation of the data; preparation, review, or approval of the manuscript; or decision to submit the manuscript for publication.
